# Low-dose spectral CTA in infant aortic coarctation

**DOI:** 10.1093/ehjcr/ytaf599

**Published:** 2026-01-12

**Authors:** Hanlin Chen, Xianfeng Chen, Shen Gui, Yanyan Jiang

**Affiliations:** Department of Radiology, Wuhan Asia General Hospital, No. 300, Taizi Lake North Road, Economic and Technological Development Zone, Wuhan, Hubei 430056, China; Department of Radiology, Wuhan Asia General Hospital, No. 300, Taizi Lake North Road, Economic and Technological Development Zone, Wuhan, Hubei 430056, China; Clinical and Technical Support, Philips Healthcare, Room 2703, Tower I, Wuhan Henglong Plaza, Qiaokou District, Wuhan, Hubei 430030, China; Department of Ultrasound, Wuhan Asia General Hospital, No. 300, Taizi Lake North Road, Economic and Technological Development Zone, Wuhan, Hubei 430056, China

**Keywords:** Spectral CT, Aortic coarctation, Infant

## Summary

A 36-day-old infant was hospitalized due to blood pressure differences between the limbs. Conventional imaging was inconclusive, but low-dose spectral computed tomography (CT) with Z-effective images clearly revealed severe coarctation of the descending aorta, later confirmed after surgery. This case shows that low-dose spectral CT angiography (CTA) can aid in accurate diagnosis and surgical planning for congenital heart disease in infants without increasing scan burden.

## Low-dose spectral CTA in infant aortic coarctation

**Figure 1:**
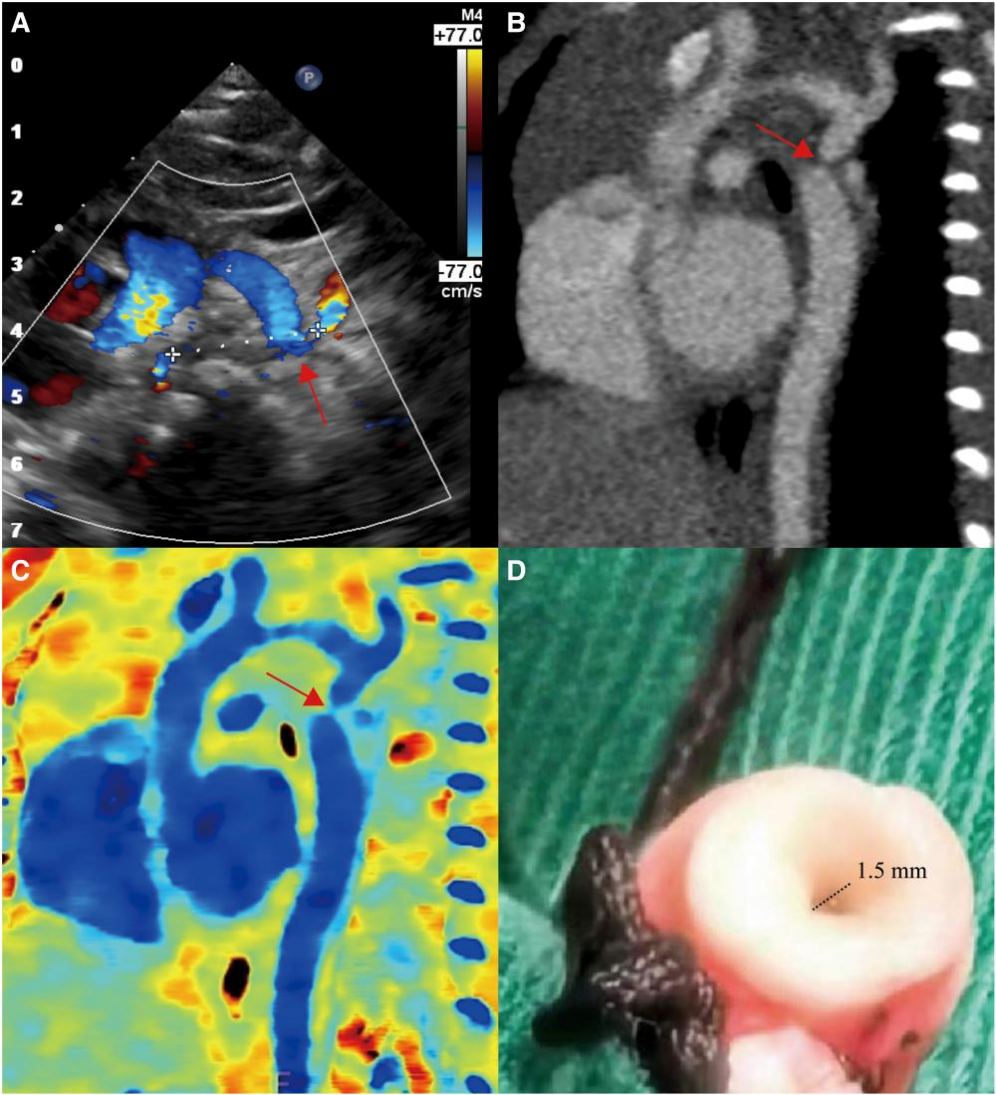
(*A*) Echocardiogram. The origin of the descending aorta, arrow. (*B*) Sagittal view of conventional CTA image. (*C*) Z-effective image. (*D*) Postoperative image of the resected stenotic segment with a diameter of 1.5 mm.

A 36-day-old infant was hospitalized after a routine checkup showed significant blood pressure differences between the upper and lower limbs. Echocardiography suggested aortic arch discontinuity (*Figure 1A*). A low-dose aortic CTA (0.39 mSv; 10 mL iohexol, 350 mg/mL) was performed using dual-layer spectral CT (Spectral CT 7500, Philips). While the conventional images suggested a possible aortic arch interruption (*Figure 1B*, red arrow), the Z-effective images clearly demonstrated severe coarctation of the descending aorta (*Figure 1C*, red arrow). Volume-rendered reconstructions from conventional and Z-effective imaging are shown in *Movies 1* and *2*, respectively. The postoperative image of the resected segment confirmed the presence of the stenosis (*Figure 1D*).

Echocardiography is the primary tool for diagnosing coarctation in infants but is limited in assessing extracardiac structures, for which CT provides high-resolution imaging. Low-dose conventional CT images failed to provide a definitive diagnosis, whereas spectral CT clearly demonstrated severe aortic coarctation on Z-effective images without increasing radiation or contrast dose^1^ Aortic coarctation is typically repaired by resecting the narrowed segment followed by end-to-end anastomosis or patch augmentation,^2^ whereas surgery for interrupted aortic arch is more complex and carries higher risks.^3^ This indicates that low-dose spectral CTA shows potential as a preferred imaging tool for infants with congenital heart disease by improving surgical planning and reducing operative risks.

## Data Availability

The data underlying this article will be shared on reasonable request to the corresponding author.

## Notes

**Consent:** Written informed consent was obtained from all subjects (patients) in this study.

## Notes

**Funding:** No funding.
